# Tracking Optical and Electronic Behaviour of Quantum Contacts in Sub-Nanometre Plasmonic Cavities

**DOI:** 10.1038/srep32988

**Published:** 2016-09-09

**Authors:** A. Sanders, R. W. Bowman, J. J. Baumberg

**Affiliations:** 1 Nanophotonics Centre, Department of Physics, Cavendish Laboratory, Cambridge, CB3 0HE, UK

## Abstract

Plasmonic interactions between two metallic tips are dynamically studied in a supercontinuum dark-field microscope and the transition between coupled and charge-transfer plasmons is directly observed in the sub-nm regime. Simultaneous measurement of the dc current, applied force, and optical scattering as the tips come together is used to determine the effects of conductive pathways within the plasmonic nano-gap. Critical conductances are experimentally identified for the first time, determining the points at which quantum tunnelling and conductive charge transport begin to influence plasmon coupling. These results advance our understanding of the relationship between conduction and plasmonics, and the fundamental quantum mechanical behaviours of plasmonic coupling.

Plasmonics, the confinement of light to nanoscale dimensions via optically-driven collective oscillations of conduction electrons, enables strong, highly-localised field enhancements. These can be exploited to realise nano-optics and nano-spectroscopy. However, the onset of quantum mechanical effects serves as a fundamental limit to plasmonic confinement in what has recently become known as the quantum regime of plasmonics^1^.

With recent technological advances, coupled plasmonic nano-cavities between metallic nanostructures with sub-nm spacings have become readily achievable, leading to both experimental^1^,^2^,^3^,^4^,^5^,^6^,^7^ as well as theoretical^8^,^9^,^10^,^11^,^12^,^13^,^14^ observations of the optical signatures of quantum mechanical effects. In particular, quantum charge transport restricts the asymptotically increasing nano-cavity field enhancement and provides a smooth transition between the classical regimes of hybridised bonding dimer plasmons (BDPs) (insulating gap) and charge transfer plasmons (CTPs) (conducting gap). Complete experimental verification directly probing the relationship between plasmon coupling and gap conductance has not yet been presented, although it is predicted to be accessible^13^,^14^.

Signatures of quantum transport have been seen in plasmonic systems using optical spectroscopy^1^,^5^,^6^, EELS^2^,^3^, SERS^6^, photoluminescence^7^, and third-harmonic generation measurements^4^. Current-induced luminescence has been explored in scanning tunnelling microscopy^15^,^16^,^17^ however no experiment to date has directly correlated all-optical measurements of plasmonic resonance behaviour with conductance. This is vital to fundamentally understand the decoupling of gap plasmons and the formation of CTPs at a critical gap separation (reported to be near the interatomic spacing of 0.3 nm^1^,^2^,^8^,^10^,^11^). It is also vital to understand why similar conductance-induced effects are also seen in larger plasmonic gaps separated by molecular spacer layers^3^,^4^,^5^,^18^. In these systems, the critical separation is simply the point at which rate of charge transfer overcomes the capacitive interaction. The implication, supported by theory^19^,^20^, is that it is a critical conductance *G*_*c*_ that controls the quantum plasmonics, rather than a critical separation as previously emphasised.

In the present work, two spherical Au-nanoparticle-tipped AFM probes approach to form sub-nm plasmonic cavities. This setup is used to dynamically investigate the quantum regime and to determine the relationship between conductance and plasmonics. By simultaneously monitoring both the electrical current passing through the dimer and its optical scattering, the effects of quantum charge transfer can be directly discerned. Using the dc conductance to track the electronic conduction across the plasmonic dimer, our experiments carefully follow its passage through the quantum regime. We develop the use of critical conductances for properly interpreting charge transfer in different plasmonic systems. Using this approach we observe the quantum mechanical limitations to plasmon coupling in full, identifying each of the key regimes of quantum plasmonic interactions more clearly than in our previous work.

## Results

**Figure 1:**
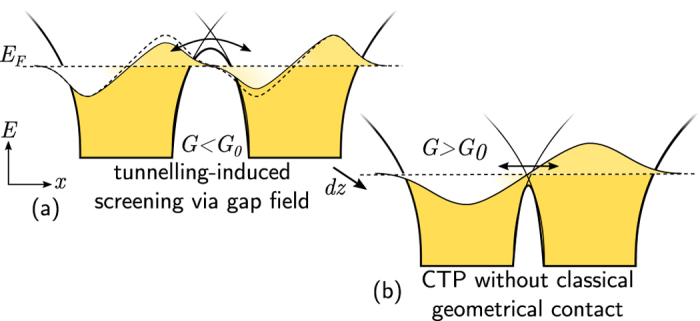
Schematic charge transfer process in a nanoparticle dimer. Electron density represented by filling of particle potential wells, with spill out from the gap surfaces. Long-range image charge interactions round off the potential barrier. (**a**) Optical-frequency t unnelling at larger distances neutralises optically-driven charge accumulation on gap surfaces, reducing coupling. (**b**) At smaller separations the particle Fermi level can become greater than the gap potential barrier, meaning the optical-frequency current bridges by conduction instead of tunnelling. This leads to gap current and CTP excitation.

Theory suggests that for separations below ~0.5 nm, within half an optical cycle the coherent electron tunnelling through the thin gap barrier fills vacant hole states at the opposite particle’s surface, neutralising the accumulated charge and reducing electromagnetic coupling, thus preventing the formation of arbitrarily large charge densities at the gap. At this separation dimers are said to be in the quantum tunnelling (crossover) regime^8^, a progressive transition into electrical contact (Fig. 1). Tunnelling thus reduces and eventually reverses the plasmonic redshifts observed, acting through screening. Below a critical gap separation of ~0.3 nm^1^,^2^,^8^,^10^,^11^ (approximately the interatomic separation) the dimer enters the CTP (conductive) regime as it passes through a critical conductivity threshold related to the conductance quantum *G*_0_ = 2 *e*^2^/*h*, where *e* is the electronic charge and *h* is Planck’s constant^21^. Hybridised gap plasmons are heavily screened and decoupled while CTP modes begin to emerge giving a blueshift of all the spectral modes. This threshold appears in time-dependent density functional theory (TDDFT) calculations once the progressively overlapping barrier edges cause the central potential barrier region to drop below the Fermi level, creating a conductive constriction or quantum point contact between surface atoms^8^ (Fig. 1). In the quantum corrected model (QCM) all effects are attributed to the increasing tunnelling current prior to conductive contact^1^,^10^,^11^.

**Figure 2:**
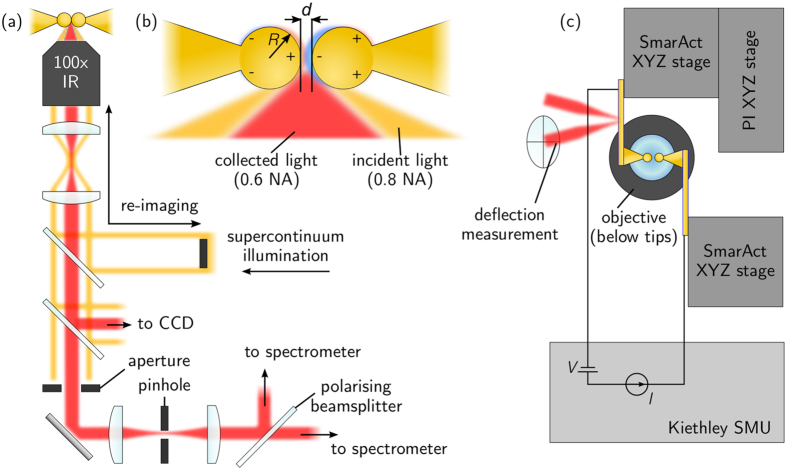
Experiment configuration for axial tip scanning. (**a**) The supercontinuum laser is centred on the aligned tip dimer for dark-field spectroscopy. Tips are illuminated at high angles to the optical axis (a central beam block is imaged into the objective’s back aperture) and scattered light is collected on-axis, using an aperture to reject reflected illumination light. The softer cantilever approaches the stationary stiffer cantilever. A bias is applied across the tip junction and the current through the gap is measured. (**b**) Diagram of tip dimer indicating separation *d*. (**c**) Mechanical configuration of the tip dimer, with electrical measurement. Applied force is measured by reflecting a laser from the softer cantilever and monitoring its deflection on a position sensitive photodiode.

We have developed a rig in which a pair of conducting AFM tips capped with spherical Au nanoparticles can be brought into sub-nm proximity to investigate the relationship between plasmonics and conductance at optical frequencies (Fig. 2). We assume that conductive behaviour originates from the same location and the same nano-scale structure at both dc and optical frequencies, with a common conductance to describe both. The tips used here are pairs of either 50 nm Au-coated Nanotools B150 AFM probes (300 nm apex diameter diamond-like-carbon tips) or similarly sized AuNP-on-Pt AFM probes fabricated in-house using electrodeposition^22^. Previously we demonstrated that tips without spherical nanoparticles (i.e. just sharp tips) do not support plasmonic cavity modes at any particular resonant wavelength but offer only broadband response^23^, hence the need to use these carefully constructed NP plasmonic probes here.

Measurements are performed in a custom-built, ultra-stable microscope utilising a supercontinuum white-light laser to spectroscopically probe single nanostructures. The microscope features integrated low-noise electronics for gap biasing and current measurements and an AFM module for force measurements. Supercontinuum laser light is spatially filtered into a ring and re-imaged onto the back-focal-plane of the objective, illuminating the focus at high angles. Low angle scatter is collected through the same objective with high angle light filtered out using an iris conjugate to the initial ring shape. This allows highly controllable dark-field spectroscopy, avoiding most light reflected from the tip surfaces and confocally imaging light from the tip gap.

The two spherical Au AFM tips are aligned in a tip-to-tip dimer configuration under the focussed laser spot using a non-linear capacitive alignment technique with the built-in AFM module^24^. The two sets of tips have different morphologies (Experimental Details). Tips are set to approach at rates of 0.1–1 nm s^−1^ whilst simultaneously recording optical scattering spectra, the current through the biased gap, and the force inferred from cantilever deflection measurements. The gap between tips is dynamically reduced and plasmons are identified from the spectrally-shifting resonances.

**Figure 3:**
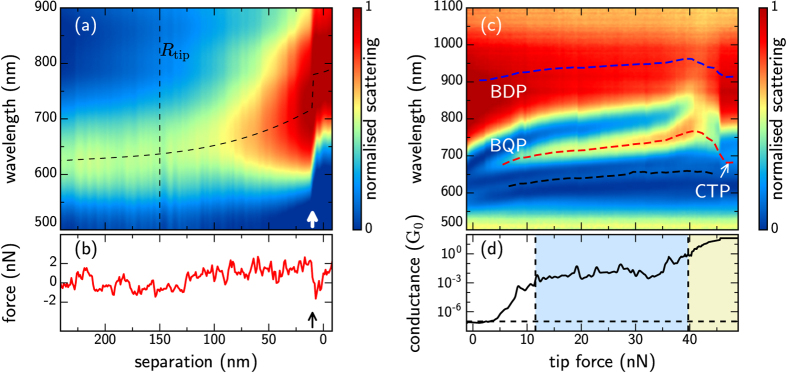
Optical scattering spectra (**a**) and force measurements (**b**) of two interacting spherical AuNP AFM tips as they are moved closer together. Dashed line shows coupling-induced redshift of the coupled BDP plasmon emerging as the separation drops below the size of the tip radius. The AFM snap-in, caused by capillary forces of gap water in ambient conditions, occurs for 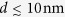 (arrows) and pulls tips together into the sub-nm regime. A similar scan at smaller separations (after snap-in) shows spectra (**c**) and conductance in units of *G*_0_ (**d**) measured as the tips are pushed through the water layer, showing quantum tunnelling (blue shaded) and conductive contact (yellow shaded).

Classical long-range capacitive coupling between plasmons is observed during approach. The single particle plasmon resonances at 630 nm from each spherical Au tip couple and redshift as the intertip separation is reduced (Fig. 3). Once the separation reduces below 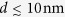 ambient water present on each tip surface forms a meniscus and capillary action pulls the tips together, known as ‘snap-in’^25^,^26^. Snap-in reduces the gap to below 1 nm and provides mechanical resistance to any further compression of the gap, which now loads force onto the cantilevers and leads to the linear reverse cantilever deflection observed in AFM force measurements. Consequently, prior to snap-in the tip displacement directly correlates with the cantilever displacement whereas after snap-in the change in tip displacement is less than the change in cantilever displacement and the applied force is used as an indication of the gap compression. This enables sub-nm gaps to be studied with some degree of control.

**Figure 4:**
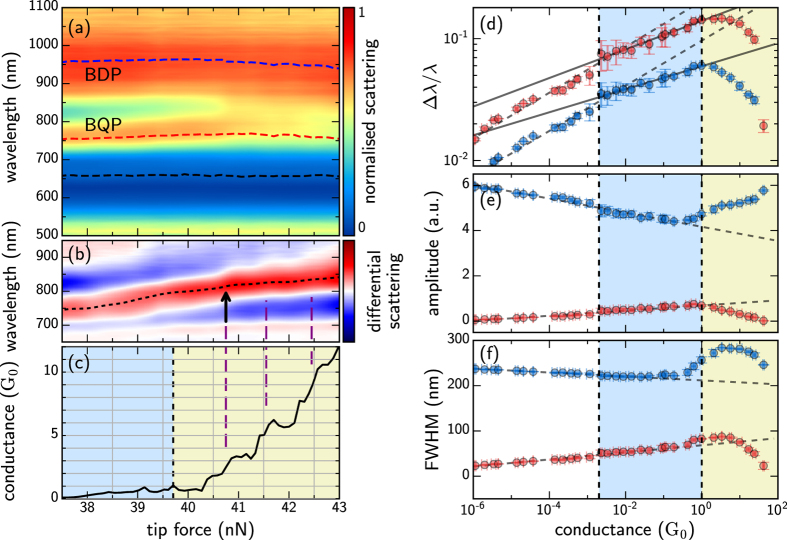
(**a**,**b**) Optical scattering spectra of a compressed sub-nm dimer cavity at the onset of conductive contact (same run as Fig. 3), showing (**c**) quantised conductance in units of *G*_0_. Deviation from the mean spectrum is shown in (b) to make the steps in spectra clearer; colour scale runs from −0.2 to 0.2 in the units of (**a**). (**d**–**f**) Extracted mode shifts, amplitudes, and linewidths for the longer wavelength (blue) and shorter wavelength (red) plasmon modes. Quantum tunnelling onset in blue shaded region, conduction in yellow shaded region.

Further compression of a plasmonic nano-cavity reduces the gap width to below 0.5 nm, leading to the onset of quantum tunnelling. A representative approach in Fig. 4(a) between two spherical Au AFM tips, shows both screening of hybridised dimer and quadrupolar plasmons (BDP, BQP) and CTP formation once in the quantum regime. Experimental plasmon modes appear at similar wavelengths to QCM predictions. The rate of redshift of each coupled mode reduces with the onset of tunnelling, along with the mode intensity, revealing that the interparticle current has risen sufficiently to begin neutralising surface charge and screening gap coupling.

Fitting these spectra and extracting the behaviour of each individual mode provides more quantitative analysis (Fig. 4d–f). Spectral mode positions approximately follow an exponential behaviour (Fig. 4d) as expected for tunnelling, and the BDP mode amplitude continually decreases, due to increasing charge localisation which reduces the outcoupling efficiency. These match recent theoretical predictions^13^,^14^. From the separation at which the spectral positions deviate from their initial exponential trends (Fig. 4d, dashed to solid line fits) we estimate a threshold conductance of *G*_*t*_ ≈ 2 × 10^−3^ *G*_0_ for entering the tunnelling regime.

Above a conductance of 1 *G*_0_, the electrical conductance clearly exhibits discrete quantised steps corresponding to unity transmission through each conductance channel in the junction (Fig. 4b), though not all steps are equally apparent (likely due to dynamics of the atomically thin wires averaging over discrete conductance states^13^,^14^). These indicate atomic contact (since the intertip separation has reached the Au interatomic separation of 0.3 nm) and the formation of an electrical contact where the Fermi level is above any remaining potential barrier.

A new behaviour is seen with a blueshifting transition between coupled plasmons and emerging CTPs occurs once the conductance exceeds *G*_*c*_ = 2 *G*_0_, signifying the rise of stronger interparticle currents and entry into the quantum conductive regime. This is in good agreement with the principles underpinning *quantum* theoretical models^8^,^27^. During this transition the intensity and width of the longest wavelength plasmon begins to increase, while the higher order plasmon attenuates into only a weak blueshifted resonance. This CTP becomes fully developed during the final atomic slip into geometrical contact. The BDP mode at this point also rapidly broadens. The fundamental CTP is expected to exist in the mid-IR, thus outside the measurable spectral range of our spectroscopy, and also predicted to be spectrally modified by the neck joint at the tip. However the behaviour of hybridised BDP, BQP modes and higher order CTPs can be clearly used to interpret the gap behaviour. It is also of note that above 2 *G*_0_, weaker echoes of the steps in conductance are observed in the optical spectra (Fig. 4b, purple dashed lines) showing that the quantisation of current in these nanojunctions can be directly observed in optics.

**Figure 5:**
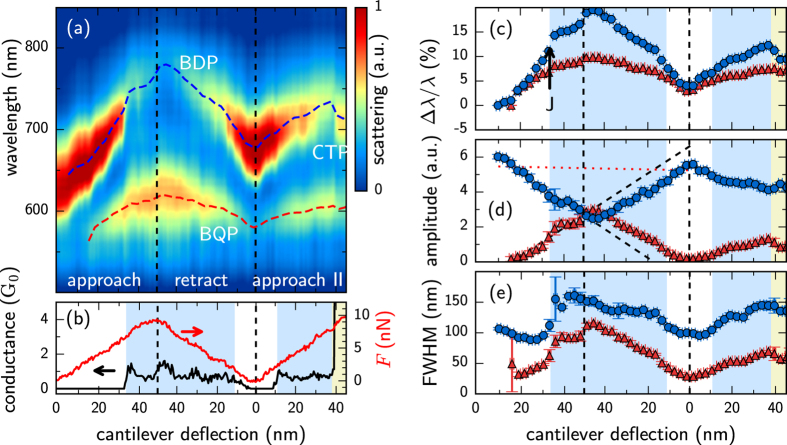
A scan showing repeated approach and retraction using electrochemically-deposited AuNP-on-Pt tips, demonstrating the reproducibility. The applied force trace in (**b**) represents separation changes between tips, with tips approached, retracted and then finally approached into contact. Peak positions in the fitted model are denoted by dashed lines superimposed on the spectra (**a**). Reduced rates of redshift are found in the *G* ≈ 1 *G*_0_ regions with a discontinuous blueshift seen after the *G* > 2 *G*_0_ transition. Linear rates of amplitude variation are revealed from peak fits after removing the width contribution from the peak intensity. As in Fig. 4, blue shaded regions represent quantum tunneling and yellow shaded regions show conductive contact. (**c**–**e**) Extracted mode shifts, amplitudes, and linewidths for the longer wavelength (blue) and shorter wavelength (red) plasmon modes.

We emphasise that the behaviour shown so far can be quite different when the nano-geometry at the tip end is modified. As a contrasting example we show a scan using highly asymmetric AuNP-on-Pt tips, smoothed using piranha solution (Fig. 5a). The extracted mode spectral shifts, mode amplitudes, and mode linewidths are shown in Fig. 5(b–e). Both cantilevers have 40 Nm^−1^ spring constants, hence the force resolution during approach is limited. In this case, prior to tunnelling (*G* < 10^−7^ *G*_0_) a higher order mode begins to emerge. The transition into contact is so quick that few intermediate points can be captured, and initially a stable 0.8–1.5 *G*_0_ contact is formed (blue shaded background in Fig. 5). As this conductance increases up to the 1 *G*_0_ level, it triggers a 4% jump in the mode wavelength (marked as J on Fig. 5(c)) and changes the rate of spectral redshifts caused by screening. The initial BDP resonance quickly weakens with the rise in conductance and the higher order resonance gains intensity. As in Fig. 4, the decreasing amplitude of the BDP mode is accompanied by a rise in the BQP mode, suggesting they are coupled depending on the gap conductivity. The screening shown here is enough to enter into the tunnelling regime but without sufficient current to enter into the conductive regime and form CTPs. Since the conductivity remains below 2 *G*_0_, no blueshift from the screening is observed.

After this first initial conductance increase, the tip is immediately retracted out of contact to test for reproducibility (vertical dashed line). This is made possible by the robustness of electrochemically fabricated tips, with their solid Au nanoparticle apices (and is not possible with the previous tips which tend to snap off at their root). A second approach of the tip immediately after retraction (right side) shows similar behaviour until the conductance rises above 2 *G*_0_. Slight differences in the redshift and amplitude show the effects of surface morphology as retraction introduces a small degree of misalignment. Once the conductance abruptly rises above *G*_*c*_ ~ 2 *G*_0_ (entering the yellow shaded region) there is a clear blueshift in the lowest order mode, again identifying the critical conductance previously found. The crossover between modes observed here is found to preserve total intensity, so that the lower mode weakens at exactly the same rate that the higher mode emerges (Fig. 5(d) shows that their sum is constant, dotted). For these plasmons, the conservation of optically-induced charge during tunnelling simply switches the favourable mode intensity as the gap width decreases.

## Discussion

Experimental identification of critical conductances is a key development in plasmonic nanogaps, distinguishing between the effects of quantum tunnelling and atomic contact. Clear blueshifting of coupled plasmons is now found to be the optical signature of atomic contact between nano-cavity surfaces. This behaviour is similar to the previously predicted classical conductance thresholds for screening and CTP excitation in linked metallic nanoparticle dimers^19^ and has implications for recent experimental systems where plasmonic surfaces are connected via molecular spacer layers^18^. Comparison with such previously explored systems shows excellent agreement, with blueshifting of the dipolar gap plasmon also observed to occur above *G*_*c*_ = 2 *G*_0_. Observation of the same threshold conductance in different experimental systems provides strong evidence for the fundamental nature of this critical conductance.

We note that the interaction of plasmonic dimers can be well modelled through *LCR* circuits^28^, producing a blue shift matching well the full classical simulations as the gap conductance is increased. This simple model predicts that the blue shift when exceeding the critical conductance is given by Δ*λ*/*λ* = *ε*_*m*_*a*/*R* where *ε*_*m*_ is the refractive index of the surrounding medium and *a* is the width of the Au cylindrical bridge between the nanoparticles compared to their radius *R*. In the situation here, we thus estimate that Δ*λ*/*λ* ~ 10% as seen in our experiments when the bridge becomes 2*a* ~ 30 nm across, confirming that indeed contacts many times larger than *G*_0_ are developed.

We note that the two examples shown here span the range of obtained spectral features, with blue shifts only resulting for conductance above *G*_*c*_, while the changes in mode intensity observed are persistent over different scans. Direct reproducibility is not yet feasible (see Experimental Section). However these results confirm that the specific atomic morphology around the plasmonic tips is crucial for the spectral evolution observed, and point to the need to control this by more sophisticated techniques.

## Conclusion

These studies show that the plasmonics of sub-nm plasmonic systems are best characterised and quantified in dedicated optomechanical rigs. Here we identify the critical conductance *G*_*c*_ allowing targeting of device geometries that can provide rapid switching. Our results also suggest that quantised conductance steps can be directly seen in purely optical measurement at room temperature in ambient conditions, through jumps in intensity and small blueshifts. Since the precise atomic-scale geometry can change on dynamical timescales currently faster than resolved with the 100 ms spectroscopy developed here, higher speed spectroscopies are essential to track the atomic movements directly in optics. Developments here should allow plasmonics to measure optical conductivities around the *G*_0_ quantum conductance level, of interest for a variety of molecular and monolayer materials, as well as enabling ultra-low energy switching in optoelectronic devices.

## Methods

**Figure 6:**
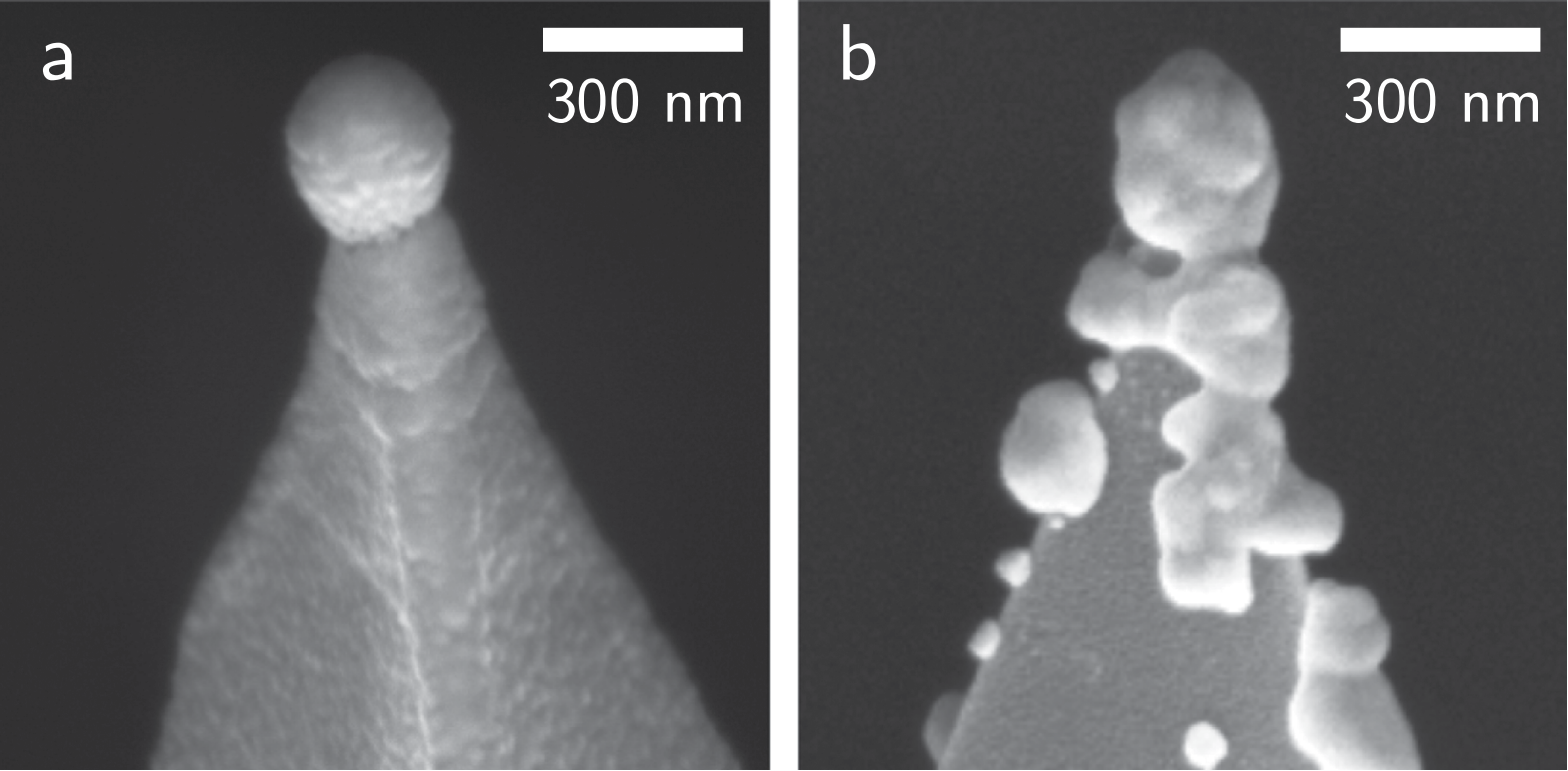
SEM images of (**a**) NanoTools spherical Au tip, and (**b**) an electrochemically deposited AuNP-on-Pt tip used in these experiments.

SEM images of the tips used in the scans presented in the main paper are shown in Fig. 6. Different tips prepared in the same way are broadly similar, but clearly differ at the nanoscale contact points. This concurs with the phenomenological experiences of making tips for tip-enhanced Raman scattering (TERS), where grains at the tip ends can have a strongly beneficial influence.

Direct reproducibility between sub-nm gaps formed in different tip dimers proves difficult as the positions and shifts of plasmon resonances can behave differently, regardless of whether they initially start when at large distances with very similar plasmonic spectral positions. We believe these differences originate from surface roughness or even atomic-scale differences in nanoscale surface morphology that affect the way optics and electronics couple^8^,^12^. Changes in CTP position and intensity are predicted to originate from the touching profile of the dimer, indicating that the surface roughness can play a massive role with such large dimer surfaces^8^,^12^. The emergence of blueshifts is less clear in many scans due to the fast transition into geometrical contact observed. This jump is experienced in almost all scans and is attributed to a combination of the electrostatic pull between tips and a sudden decrease in mechanical resistance once the water molecules in the gap are displaced allowing the mobile gold atoms to jump the gap. The approach shown in Fig. 4 is carefully controlled going into geometrical contact and provides a much clearer insight into the origins of the blueshift.

### Data availability.

Additional data related to this publication is available at the University of Cambridge data repository (http://dx.doi.org/10.17863/CAM.1309).

## Additional Information

**How to cite this article**: Sanders, A. *et al.* Tracking Optical and Electronic Behaviour of Quantum Contacts in Sub-Nanometre Plasmonic Cavities. *Sci. Rep.* **6**, 32988; doi: 10.1038/srep32988 (2016).
